# From the Cochrane Library: Dietary Supplements for Established Atopic Eczema

**DOI:** 10.2196/33178

**Published:** 2022-07-29

**Authors:** Madeline J Adelman, Torunn E Sivesind, Robert P Dellavalle

**Affiliations:** 1 Department of Dermatology University of Colorado Anschutz Medical Campus Aurora, CO United States; 2 Department of Epidemiology Colorado School of Public Health University of Colorado Anschutz Medical Campus Denver, CO United States; 3 Dermatology Service Department of Veterans Affairs Medical Center Denver, CO United States

**Keywords:** atopic dermatitis, dietary supplements, atopy, fish oil, systemic review, atopic eczema, eczema, probiotics

Atopic eczema (AE) is a chronic inflammatory skin condition, affecting 5% to 20% of people worldwide [1]. While there are many available treatment options that help improve AE, patients for whom these treatments did not work, or who fear side effects, may look to nutritional supplements as “natural” solutions. Nutritional supplements represent a vast, growing industry, globally valued at over US $150 billion in 2021. Nutritional supplements are manufactured pills, powders, or liquids, meant to provide nutrients in addition to conventional food. Supplements are classified by the Food and Drug Administration as foods rather than drugs; therefore, they are not required to prove efficacy or safety prior to entering the market. Given the growing popularity of supplements, physicians must be knowledgeable about supplement ingredients when counseling patients. A 2012 Cochrane review, “Dietary supplements for established atopic eczema” [1], offers a comprehensive review of evidence regarding popular dietary supplements used in AE. Here, we discuss the findings of this Cochrane review and of relevant subsequent publications. Of note, although food allergies often coexist in patients with AE, supplements were studied for their effects on AE and not as treatments for food allergies.

The review [1] extracted data and assessed the quality of 11 randomized controlled trials, with a total of 596 participants, investigating therapeutic interventions of fish oil, zinc, selenium, vitamin D, vitamin E, vitamin B6, sea buckthorn oil, hempseed oil, and sunflower oil, versus placebo. Participants had physician-diagnosed AE, with 8 studies using the Hanifin and/or Rajka criteria; the other 3 studies did not state a diagnostic method. The authors evaluated evidence of symptom improvement in the short term, reduced number of flares in the long term, and a reduced need for treatment in the long term.

Overall, there was scarce evidence supporting the use of supplements for treating AE. However, given that many of the included studies were either underpowered or of low quality, evidence was insufficient to claim all supplements are completely ineffective. Nevertheless, the authors advised against further research without a stronger rationale—to the exclusion of fish oil, for which pooled data from 2 small studies suggest it may improve subjective daily quality of life in people with AE [1]. Further research on fish oil is warranted, with preliminary evidence suggesting it may down-regulate inflammation. A 2018 animal study demonstrated that n-3 polyunsaturated fatty acids depressed inflammasome activation, with a resultant reduction in inflammatory cytokine release and overall inflammatory response, as well as marked attenuation of atopic skin lesions [2]. Additionally, a 2018 cross-sectional survey found that 35% of patients who added fish oil to their diet reported an improvement in their AE symptoms [3].

In a 2018 review of probiotics, vitamins, oils, and traditional Ayurvedic agents*,* there was insufficient evidence to recommend any oral supplements as treatment, with the exclusion of probiotics [4]. Meta-analyses of probiotics have produced conflicting results; variable patient populations, probiotic strains, dosing, and duration of therapy among studies limit the pooling of data for a meta-analysis. Additional large-scale clinical trials are necessary to fully understand the benefits of probiotics for AEs, elucidate optimal strains, and determine which patient populations would achieve the greatest benefit.

Overall, this review highlights the limited evidence that exists for the use of nutritional supplements in AE. At present, the most effective “natural” modality for AE is topical emollients [5]. Future randomized controlled trials of promising dietary supplements should include patient-reported outcomes to fully assess the impact of nutritional modifications.

## Abbreviations

AE: atopic eczema
